# Intervenção Coronária Percutânea com Volume de Contraste Ultrabaixo versus Intervenção Coronária Percutânea Convencional em Pacientes com Insuficiência Renal Pré-Existente: Uma Revisão Sistemática e Metanálise de Desfechos Clínicos

**DOI:** 10.36660/abc.20250660

**Published:** 2026-05-15

**Authors:** Marcos Danillo Oliveira, Ricardo Fonseca Oliveira Suruagy-Motta, Leonardo Dexheimer da Silva, Karlos Daniell Araújo dos Santos, Adriano Caixeta

**Affiliations:** 1 Departamento de Cardiologia Intervencionista Universidade Federal de São Paulo São Paulo SP Brasil Departamento de Cardiologia Intervencionista, Universidade Federal de São Paulo, São Paulo, SP – Brasil; 2 Departamento de Medicina Universidade Cesmac Maceió AL Brasil Departamento de Medicina, Universidade Cesmac, Maceió, AL – Brasil; 3 Departamento de Medicina Universidade de São Paulo São Paulo SP Brasil Departamento de Medicina, Universidade de São Paulo, São Paulo, SP – Brasil; 4 Departamento de Medicina Universidade Federal de Roraima Boa Vista RR Brasil Departamento de Medicina, Universidade Federal de Roraima, Boa Vista, RR – Brasil

**Keywords:** Meios de Contraste, Intervenção Coronária Percutânea, Injúria Renal Aguda, Revisão Sistemática, Metanálise.

## Abstract

**Fundamento:**

A lesão renal aguda associada ao contraste (LRA-AC) resulta em aumento da morbidade e mortalidade, hospitalização prolongada e piores desfechos clínicos. Estudos anteriores demonstraram que limitar o volume de contraste (VC) a menos de 3 vezes a taxa de filtração glomerular estimada (TFGe) implica menor risco de LRA-AC, e tanto a eficácia quanto os melhores desfechos estão associados a procedimentos coronários com volume de contraste ultrabaixo (VCU) (VC total ≤ TFGe).

**Objetivos:**

Esta revisão sistemática e metanálise teve como objetivo comparar os desfechos clínicos da intervenção coronária percutânea (ICP) com VCU versus ICP convencional em pacientes com doença renal crônica.

**Métodos:**

A busca foi realizada nas bases de dados PubMed/MEDLINE, Cochrane, Embase, Scopus e Web of Science, incluindo todos os estudos que relataram desfechos clínicos de ICP VCU. O desfecho primário de eficácia foi a incidência de lesão renal aguda associada à quimioterapia (LRA-AC), e os desfechos secundários foram a necessidade de diálise, mortalidade por todas as causas e eventos cardiovasculares adversos maiores (MACE), definidos como o desfecho composto de mortalidade por todas as causas, infarto do miocárdio não fatal e revascularização da lesão-alvo clinicamente indicada.

**Resultados:**

Seis estudos atenderam aos critérios de inclusão e foram incluídos na análise final, abrangendo um total de 274.102 pacientes. A ICP VCU foi associada à redução estatisticamente significativa da LRA-AC (razão de risco = 0,27; IC 95% = 0,13–0,56; p = 0,0004) que foi observada. Por outro lado, a ICP VCU não resultou em redução estatisticamente significativa da necessidade de diálise (razão de risco = 0,33; IC 95% = 0,06–1,72; p = 0,186), mortalidade por todas as causas (razão de risco = 0,48; IC 95% = 0,17–1,39; p = 0,18) e eventos cardiovasculares adversos maiores (MACE) (razão de risco = 0,52; IC 95% = 0,21–1,30; p = 0,16).

**Conclusões:**

A ICP VCU, em comparação com a ICP convencional, em pacientes com doença renal crônica, se mostrou segura, viável e associada a uma redução estatisticamente significativa da LRA-AC. Ensaios adicionais, de maior porte, randomizados e controlados, são necessários para confirmar essas descobertas.

## Graphical abstract

**Figura Central:**
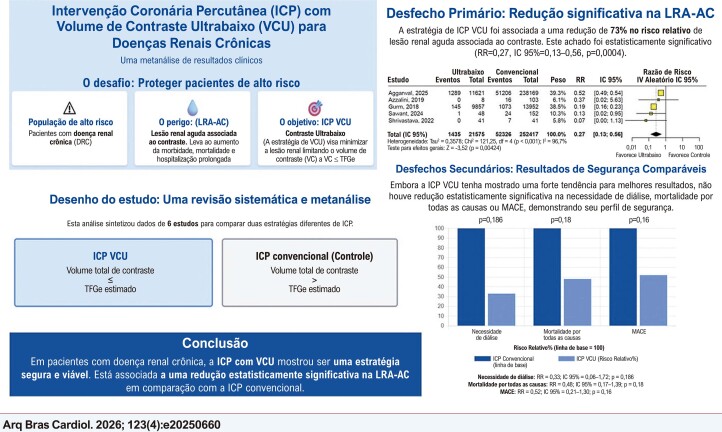
Intervenção Coronária Percutânea com Volume de Contraste Ultrabaixo versus Intervenção Coronária Percutânea Convencional em Pacientes com Insuficiência Renal Pré-Existente: Uma Revisão Sistemática e Metanálise de Desfechos Clínicos

## Introdução

Caracterizada por uma disfunção renal que ocorre poucos dias após a administração intravascular de contraste, na ausência de outras etiologias,^1,2^ a lesão renal aguda associada ao contraste (LRA-AC) resulta em aumento da morbidade e da mortalidade, hospitalização prolongada e piores desfechos clínicos.^3-5^

Ao longo do tempo, o melhor reconhecimento dos fatores de risco, a otimização dos agentes de contraste e a implementação de cuidados preventivos resultaram em taxas mais baixas de LRA-AC.^2^A hidratação adequada, o uso de meios de contraste de baixa osmolaridade ou iso-osmolaridade e a minimização do volume de contraste (VC) são os únicos recursos baseados em evidências que se mostraram eficazes na redução da LRA-AC.^3,6,7^

Estudos anteriores demonstraram que limitar o VC a menos de 3 vezes a taxa de filtração glomerular estimada (TFGe) implica menor risco de LRA-AC,^6,8,9^ e tanto a eficácia quanto os melhores resultados estão associados a procedimentos coronários com volume de contraste ultrabaixo (VCU) (VC total ≤ TFGe).^6,10-13^

Esta revisão sistemática e meta-análise teve como objetivo comparar os resultados clínicos da ICP VCU versus a intervenção coronária percutânea (ICP) convencional em pacientes com doença renal crônica (DRC).

## Métodos

Esta revisão sistemática com meta-análise seguiu as diretrizes e resoluções determinadas pelo Manual Cochrane^14^e a PRISMA de 2020.^15^Além disso, este projeto foi registrado no PROSPERO (https://www.crd.york.ac.uk/PROSPERO/) com o número de protocolo CRD420251108750.

### Estratégia de busca

A busca foi realizada de forma autônoma por dois pesquisadores (RS-M e CF) nas bases de dados PubMed/MEDLINE, Cochrane, Embase, Scopus e Web of Science, incluindo todos os estudos que relataram desfechos clínicos de ICP VCU. Os operadores booleanos “AND” e “OR” foram aplicados para combinar os termos “*Ultra-Low Contrast*” (Contraste Ultrabaixo), “*Percutaneous Coronary Intervention*” (Intervenção Coronária Percutânea) e “*PCI*” (Intervenção Coronária Percutânea). Essa mesma estratégia foi utilizada em todas as bases de dados.

Critérios de elegibilidade

#### Critérios de inclusão

A principal questão desta revisão sistemática foi: “A ICP com contraste ultrabaixo, em comparação com a ICP convencional, é segura e viável?” Esta questão foi fundamentada e formulada utilizando a estrutura PICOTT,^16,17^ com os seguintes componentes:

- **População (P):** adultos com DRC basal (TFGe < 60 mL/min/ 1,73 m^2^) submetidos a ICP;
- **Intervenção (I):** ICP de contraste ultrabaixo (VC total ≤ TFGe para cada paciente respectivo);
- **Comparação (C):** ICP convencional (após opacificação sistemática padrão das artérias coronárias com contraste para planejamento, orientação e avaliação de múltiplas etapas do procedimento e resultados da ICP, sem tentativas, técnicas ou estratégias para minimizar o VC total);
- **Resultados (O):**

– Desfecho primário: LRA-AC peri-procedural;

– Desfechos secundários: necessidade de diálise, mortalidade por todas as causas e eventos cardiovasculares adversos graves (MACE), definidos como o desfecho composto de mortalidade por todas as causas, infarto do miocárdio não fatal e revascularização da lesão-alvo clinicamente indicada;

- **Tipo de estudo (T):** ensaios clínicos randomizados e estudos observacionais;
- **Tempo (T):** Acompanhamento perioperatório de até 6 meses.

#### Critérios de exclusão

Os critérios de exclusão aplicados neste estudo foram os seguintes: 1) estudos que abordaram apenas a estratégia VCU sem nenhum grupo de comparação; 2) estudos que relataram exclusivamente os resultados da ICP guiada por ultrassom intravascular (IVUS); 3) estudos sem relato explícito de VC; e 4) estudos que não relataram dados quantitativos sobre LRA-AC ou desfechos renais relevantes. Além disso, relatos de caso, revisões, resumos de congressos e pesquisas não originais não foram incluídos nesta revisão sistemática e meta-análise.

## Seleção de estudos

A primeira triagem teve como alvo principal o título e o resumo, sem restrições quanto à data de publicação. Todos os artigos foram importados para a plataforma Rayyan (https://www.rayyan.ai/).^18^ Inicialmente, todas as duplicatas foram excluídas e, em seguida, se iniciou a triagem de títulos e resumos. Nessa etapa, todos os artigos que não se relacionavam diretamente ao tema de interesse foram excluídos. Essa etapa foi realizada independentemente por dois revisores (RS-M e LS); quaisquer dúvidas foram resolvidas com o auxílio de um terceiro pesquisador (MDO).

## Pontos finais

O desfecho primário de eficácia foi a incidência de LRA-AC periprocedural.

Os desfechos secundários foram a necessidade de diálise, a mortalidade por todas as causas e o MACE, definido como o desfecho composto de mortalidade por todas as causas, infarto do miocárdio não fatal e revascularização da lesão-alvo clinicamente indicada, no período perioperatório, com acompanhamento de até 6 meses.

## Definições

A ICP VCU foi definida como a ICP realizada utilizando um VC total ≤ TFGe para cada paciente.

A ICP zero contraste (ou sem contraste), um subconjunto especial da ICP VCU, foi definida como a ICP realizada utilizando zero mL de contraste.

A ICP convencional foi definida como a ICP realizada com opacificação sistemática padrão das artérias coronárias por contraste para planejamento, orientação e avaliação de múltiplas etapas do procedimento e resultados da ICP, sem tentativas, técnicas ou estratégias para minimizar o risco cardiovascular total.

De acordo com a *Kidney Disease Improving Global Outcomes* (KDIGO), a LRA-AC foi definida como um aumento na creatinina sérica de ≥0,3 mg/dL em até 48 horas após a exposição ao meio de contraste, ou de ≥50% em até 7 dias.^19^

## Extração de dados

Os seguintes dados foram extraídos dos artigos selecionados de acordo com os critérios do estudo: autores, ano de publicação, local do estudo, tipo de estudo (ensaio clínico randomizado ou observacional), tamanho da amostra, características demográficas dos pacientes, características do procedimento, VC, tempo de seguimento, desfechos e resultados (LRA-AC, necessidade de diálise, mortalidade por todas as causas e MACE) e principais resultados. Dois revisores (RS-M e LS) extraíram e gerenciaram todos os dados de forma independente, os quais foram registrados em uma planilha do Excel^®^. Quaisquer dúvidas foram esclarecidas com o auxílio de um terceiro pesquisador (MDO).

## Avaliação de qualidade

Uma metanálise incluiu um ensaio clínico randomizado e cinco estudos observacionais, além da ferramenta ROBINS-I (*Risk Of Bias In Non-randomized Studies of Interventions*).^20^A metodologia aplicada focou especialmente no viés relacionado ao desenho do estudo, fatores de confusão, seleção dos participantes e avaliação dos desfechos. Dois revisores independentes (LS e RS-M) realizaram as avaliações de risco de viés, atribuindo pontuações com base em critérios estabelecidos para cada instrumento. As discrepâncias entre os revisores foram resolvidas por meio de discussão e, quando necessário, um terceiro revisor (MDO) foi consultado para chegar a um consenso. Os autores não utilizaram nenhuma ferramenta automatizada durante a avaliação do risco de viés; todas as classificações e avaliações foram realizadas manualmente por revisores experientes.

## Análise estatística

Esta meta-análise foi conduzida seguindo padrões metodológicos estabelecidos. Um modelo de efeitos aleatórios foi empregado para levar em consideração a potencial heterogeneidade entre os estudos. Para desfechos binários, a razão de risco (RR) com o respectivo intervalo de confiança (IC) de 95% foi calculada. A significância estatística foi definida como valores de p < 0,05. A heterogeneidade foi avaliada utilizando a estatística I^2^, com valores > 50% indicando heterogeneidade substancial. Nesses casos, análises de sensibilidade foram realizadas utilizando o método *leave-one-out*, e gráficos de Baujat foram gerados para avaliar a influência de cada estudo individual no tamanho do efeito geral. O potencial viés de publicação foi avaliado por meio de gráficos de funil construídos para cada desfecho. Todas as análises estatísticas foram conduzidas utilizando o R^™^ no ambiente RStudio^™^ (versão 2024.11.0+488).

## Resultados

### Seleção de estudos

**Figura 1:**
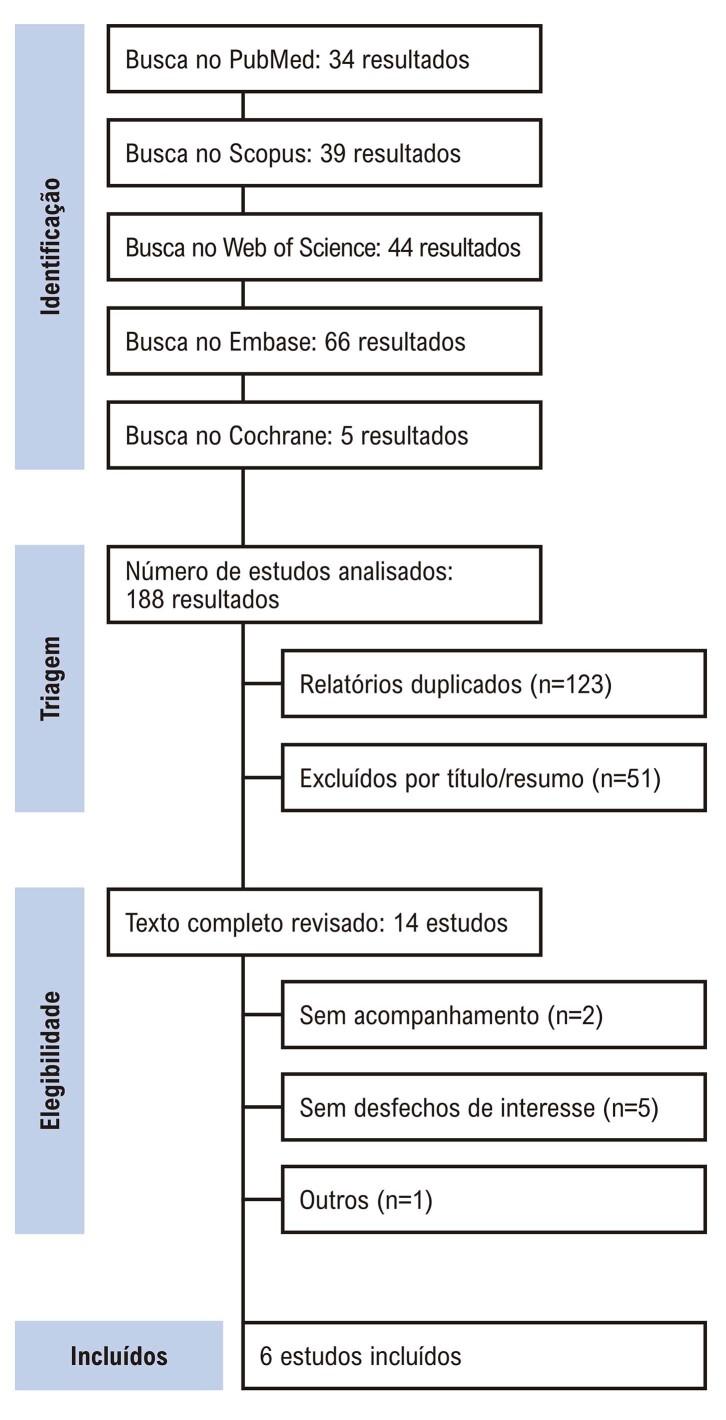
– Fluxograma PRISMA.

Um total de 188 registros foram identificados no PubMed (n = 34), Scopus (n = 39), Web of Science (n = 44), Embase (n = 66) e Cochrane (n = 5). Após a remoção de 123 duplicatas, 65 registros permaneceram para a triagem de título e resumo. Destes, 14 artigos foram selecionados para avaliação do texto completo. Finalmente, seis estudos atenderam aos critérios de inclusão e foram incluídos na análise final. Esse processo de busca está ilustrado na Figura 1.

### Características basais dos estudos incluídos e dos pacientes

A Tabela 1 apresenta as características dos estudos incluídos na presente revisão sistemática e metanálise, descrevendo as informações basais dos pacientes. A maioria dos estudos demonstrou bom equilíbrio entre os dois grupos em relação ao número de participantes, distribuição por sexo e idade. No entanto, houve algumas variações importantes nos valores de TFGe e na prevalência de comorbidades.

**Tabela 1:** – Características dos estudos incluídos na presente revisão sistemática e metanálise, descrevendo as informações basais dos pacientes

Autor, ano	País	Desenho do estudo	Número total de pacientes		Macho		Idade média		Comorbidades		Doença renal crônica		Taxa de filtração glomerular estimada (mL/min/1,73 m^2^)		Volume total de contraste (mL)		Número de stents implantados	
-	-	-	IPC Contraste Ultrabaixo	IPC Convencional	IPC Contraste Ultrabaixo	IPC Convencional	IPC Contraste Ultrabaixo	IPC Convencional	IPC Contraste Ultrabaixo	IPC Convencional	IPC Contraste Ultrabaixo	IPC Convencional	IPC Contraste Ultrabaixo	IPC Convencional	IPC Contraste Ultrabaixo	IPC Convencional	IPC Contraste Ultrabaixo	IPC Convencional
Shrivastava et al.^29^	Índia	Aleatorizado	41	41	31 (76)	30 (73)	61,44 ± 8,8	60,17 ± 8,17	34 (83) Diabetes / 28 (68) Hipertensão	32 (78) Diabetes / 36 (88) Hipertensão	26 (63,4)	26 (63,4)	42,20 ± 9,96	41,87 ± 9,34	41,02 (±9,8)	112,54 ± 25,18	1,46 ± 0,66	1,71 ± 0,71
Shibata et al.^21^	Japão	Coorte	50	50	44 (88)	43 (87)	72,3 ± 12,4	70,7 ± 12,0	27 (54,0) Diabetes / 35 (70,0) Hipertensão	29 (58,0) Diabetes / 34 (68,0) Hipertensão	50 (100)	50 (100)	38,9 ± 15,2	72,6 ± 13,6	4,4 ± 1,3 (IPC)	86,4 ± 38,9 (IPC)	1,14 ± 0,076	1,27 ± 0,07
Sawant et al.^31^	EUA	Retrospectivo	48	152	39 (81)	110 (72)	69 ± 9	68 ± 10	26 (54,2) Diabetes / 44 (91,7) Hipertensão	74 (48,7) Diabetes / 147 (96,7) Hipertensão	NR	NR	NR	NR	19,17 ± 7,29	147,14 ± 73,55	NR	NR
Gurm et al.^6^	EUA	Retrospectivo	9.857	13.952	6872 (69,7)	8017 (57,5)	57,49 ± 10,85	74,38 ± 10,46	4229 (42,9) Diabetes / 8181 (83,0) Hipertensão	5436 (39,0) Diabetes / 12 534 (89,9) Hipertensão	NR	NR	NR	NR	106,58 ± 41,19	228,61 ± 83,76	NR	NR
Aggarwal et al.^9^	EUA	Coorte	11.621	238.169	7391 (63,6)	139.329 (58,5)	73,7 ± 11,1**	73,7 ± 11,1**	6.461 (55,6) Diabetes / 10.842 (93,3) Hipertensão	128.135 (53,8) Diabetes / 219.592 (92,2) Hipertensão	11.621 (100)	238.169 (100)	NR	NR	CV/eGFR < 1	CV / eGFR > 3	NR	NR
Azzalini et al.^4^	Itália	Retrospectivo	8	103	83 (75)		76,0 ± 7,4**		62 (56) Diabetes		8 (100)	103 (100)	19,7 ± 4,4**	24,8 ± 5,9**	9,5 ± 12,7**	96,0 ± 60,7**	NR	NR

Observação: Os dados são apresentados como média ± desvio padrão, número (nº) ou mediana (intervalo interquartil); IPC: intervenção coronária percutânea; NR: não relatado. *Valores apresentados como N (%) ou média ± desvio padrão; **Valores transformados em média ± desvio padrão usando o método de Wan et al.^*24*^

### Desfecho primário

#### Lesão renal aguda associada ao contraste

**Figura 2:**
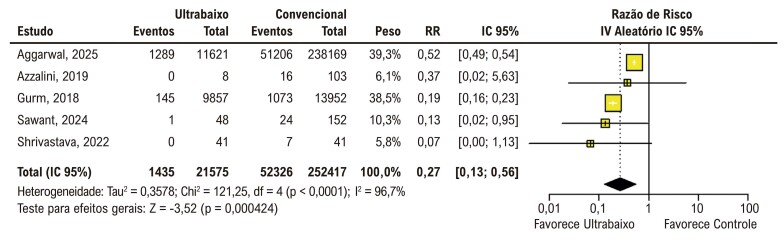
– Gráfico de floresta para o desfecho de LRA-AC. A análise mostra as taxas gerais de LRA-AC comparando ICP VCU versus ICP convencional nos estudos incluídos. O losango preto na parte inferior representa o RR agrupado derivado da metanálise. LRA-AC: lesão renal aguda associada ao contraste; VCU: contraste ultrabaixo; ICP: intervenção coronária percutânea; IC: intervalo de confiança; RR: razão de risco.

A metanálise da incidência de LRA-AC demonstrou um RR agrupado geral de 0,27 (IC 95% = 0,13–0,56) sob um modelo de efeitos aleatórios, favorecendo a ICP VCU em relação à ICP convencional. A análise revelou heterogeneidade substancial (I^2^ = 96,7%), indicando variabilidade significativa entre os estudos incluídos em termos de desenho, populações e protocolos de procedimento. Apesar dessa alta heterogeneidade, o teste para o efeito geral foi estatisticamente significativo (Z = -3,52, p = 0,0004), apoiando um potencial papel protetor da ICP VCU na redução da LRA-AC, em comparação com a ICP convencional. Figura 2 e Figura Central).

## Resultados secundários

### Necessidade de diálise

**Figura 3:**
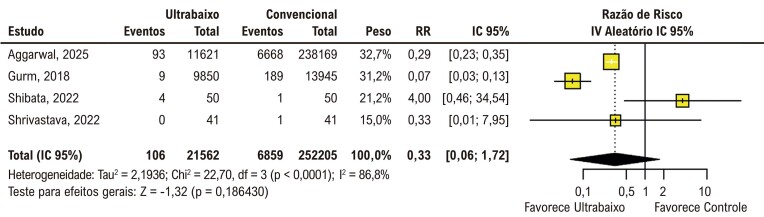
– Gráfico de floresta para o desfecho de necessidade de diálise. A análise mostra as taxas gerais de necessidade de diálise comparando ICP VCU versus ICP convencional nos estudos incluídos. O losango preto na parte inferior representa o RR agrupado derivado da metanálise. VCU: contraste ultrabaixo; ICP: intervenção coronária percutânea; IC: intervalo de confiança; RR: razão de risco.

A metanálise da necessidade de diálise demonstrou um RR agrupado geral de 0,33 (IC 95% = 0,06–1,72) sob um modelo de efeitos aleatórios, favorecendo a ICP VCU, em comparação com a ICP convencional, embora sem significância estatística (Z = −1,32, p = 0,186). A análise revelou heterogeneidade considerável (I^2^ = 86,8%), sugerindo diferenças substanciais entre os estudos incluídos em termos de desenho, função renal basal e critérios para início da terapia de substituição renal. Embora a estimativa pontual sugira um potencial efeito protetor, o amplo intervalo de confiança e a alta heterogeneidade impedem conclusões definitivas. Figura 3 e Figura Central).

### Mortalidade por todas as causas

**Figura 4:**
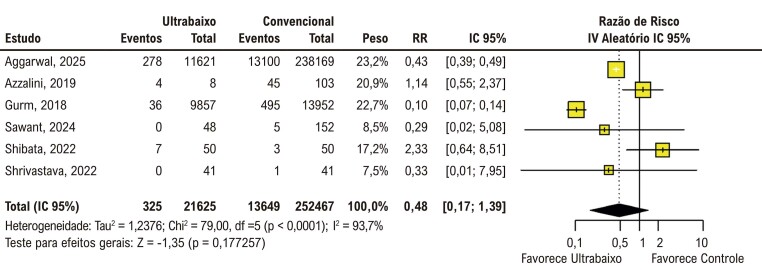
– Gráfico de floresta para o desfecho de mortalidade por todas as causas. A análise mostra as taxas de mortalidade geral comparando a ICP VCU versus ICP convencional em todos os estudos incluídos. O losango preto na parte inferior representa o risco relativo (RR) agrupado.

A metanálise da mortalidade por todas as causas demonstrou um RR agrupado geral de 0,48 (IC 95% = 0,17–1,39) sob o modelo de efeitos aleatórios, favorecendo a ICP VCU em relação à ICP convencional, embora sem atingir significância estatística (Z = -1,35, p = 0,18). O alto grau de heterogeneidade (I^2^ = 93,7%) indica variabilidade substancial entre os estudos incluídos, provavelmente devido a diferenças na seleção de pacientes, técnicas de procedimento ou definições de desfecho. Embora a estimativa pontual sugira um potencial benefício na mortalidade para estratégias VCU, o amplo IC e a marcada heterogeneidade impedem conclusões confiáveis. Figura 4 e Figura Central).

### Eventos cardiovasculares adversos graves

**Figura 5:**
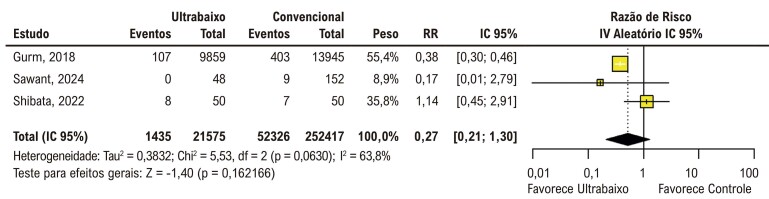
– Gráfico de floresta para o desfecho MACE. A análise mostra as taxas gerais de MACE comparando ICP VCU versus ICP convencional nos estudos incluídos. O losango preto na parte inferior representa o RR agrupado derivado da metanálise. MACE: desfecho cardiovascular adverso maior; VCU: contraste ultrabaixo; ICP: intervenção coronária percutânea; IC: intervalo de confiança; RR: razão de risco.

A metanálise de eventos cardiovasculares adversos maiores (MACE) mostrou um risco relativo (RR) agrupado geral de 0,52 (IC 95% = 0,21–1,30) sob o modelo de efeitos aleatórios, sugerindo uma possível redução de MACE com a ICP VCU, em comparação com a ICP convencional, embora esse achado não tenha atingido significância estatística (Z = -1,40, p = 0,16). Observou-se heterogeneidade moderada (I^2^ = 63,8%), refletindo alguma variabilidade nos protocolos dos estudos, nas definições de MACE ou nas populações de pacientes. Apesar da direção do efeito favorecer a ICP VCU, o IC foi amplo e cruzou a unidade, impedindo conclusões definitivas. Figura 5 e Figura Central).

## Análise de sensibilidade

Foram realizadas análises de sensibilidade utilizando os métodos *Leave-one-out*, Baujat e Funil para investigar as fontes de heterogeneidade. A exclusão de Shibata et al.^21^reduziu a heterogeneidade para 0% para o desfecho MACE (Figura 5), e a exclusão de Gurm et al.^8^ reduziu a heterogeneidade para 68% para mortalidade por todas as causas (Figura 4) e para 65% para necessidade de diálise, respectivamente (Figura 3).

## Avaliação da qualidade

Um ensaio randomizado foi incluído nesta metanálise, e a avaliação do risco de viés (RoB) foi realizada por meio da ferramenta Cochrane RoB 2,^22^ seguindo o Manual Cochrane para Revisões Sistemáticas de Intervenções,^14^ o que indicou um risco moderado de viés. Isso se deveu principalmente a métodos de randomização pouco claros e à falta de alguns dados de desfecho (Figura Suplementar 1).

Adicionalmente, cinco estudos não randomizados foram incluídos nesta metanálise e avaliados pela ferramenta ROBINS-I^20^(Figura Suplementar 1). Dois desses estudos apresentaram um risco sério de viés, principalmente devido a diferenças basais entre os dois grupos analisados: houve diferenças significativas na TFGe e disparidades substanciais no número de pacientes em cada grupo. Esses desequilíbrios basais podem introduzir fatores de confusão que podem distorcer os efeitos observados e comprometer a validade interna.

Outros estudos demonstraram um risco moderado de viés no Domínio 1 (fatores de confusão). Enquanto isso, a ferramenta ROBINS-I^20^embora forneça uma abordagem estruturada para avaliar esse domínio, o risco inerente aos estudos não randomizados deve ser considerado, e alguns fatores de confusão residuais ainda podem influenciar os resultados.

Outros riscos identificados incluíram protocolos de estudo pouco claros e alocação retrospectiva de pacientes, ambos capazes de introduzir vieses. Protocolos pouco claros podem levar a inconsistências na implementação do estudo, enquanto a alocação retrospectiva pode resultar em viés de seleção.

## Meta-regressão

Devido à elevada heterogeneidade observada, foi realizada uma análise de meta-regressão para explorar possíveis fontes de variabilidade. Foi encontrada uma associação estatisticamente significativa entre a idade média dos pacientes no grupo ICP VCU e os riscos de LRA-AC ( (log⁡[RR]=−5,20+0,0615x idade média, p < 0,001  , p < 0,001, R^2^ = 100%, Figura Suplementar 2) e morte ( log⁡[RR]=−9,11+0,1205× idade média  , p = 0,009, R^2^ = 66,8%, I^2^ residual 69%, Figura suplementar 3), indicando que a idade explicou a heterogeneidade entre os estudos. Este achado pode ser explicado pela conhecida relação entre idade e reserva funcional reduzida, sugerindo que indivíduos mais velhos têm maior probabilidade de desenvolver LRA-AC e de morrer, em comparação com os mais jovens.

## Discussão

A principal conclusão desta revisão sistemática e metanálise que comparou a ICP VCU com a ICP convencional em pacientes com doença renal crônica (DRC) basal foi que a ICP VCU resultou em uma estatisticamente significativa redução (RR=0,27; IC 95%=0,13–0,56; p=0,0004) de LRA-AC, que está associada a prognóstico desfavorável, desfechos clínicos deletérios, hospitalização prolongada e aumento da mortalidade.^3-5^

A ICP VCU é uma abordagem contemporânea que incorpora as melhores práticas, como fisiologia invasiva, imagem intravascular e ferramentas radiográficas avançadas em vez de contraste radiográfico.^4^Ao combinar essas ferramentas, com a esperada melhoria na segurança, os resultados dos procedimentos, tanto a curto quanto a longo prazo, devem ser melhores do que com a orientação angiográfica convencional.^5^

Atualmente, são descritas diversas estratégias para reduzir o VC durante a ICP.^5^Dentre essas abordagens, a orientação por IVUS parece ser a mais eficaz.

O estudo MOZART (*Minimizing cOntrast utiliZation With IVUS Guidance in coRonary angioplasTy*)^23^foi o primeiro estudo randomizado a comprovar que uma estratégia de imagem intravascular prévia pode reduzir o uso de contraste em cerca de 2/3 sem prejudicar os resultados da ICP aguda. Kumar et al.^24^descreveram sua experiência com ICP guiada por IVUS sem contraste em 42 pacientes consecutivos com DRC (TFGe média: 30,67 ± 12,26 mL/min/1,73 m^2^, 66 vasos, 21,2% ICP na artéria coronária esquerda principal). O sucesso técnico foi de 92,4%, sem complicações maiores, durante o acompanhamento de 3 a 12 meses. Truong et al.^25^avaliaram uma estratégia de ICP VCU guiada por IVUS em 80 pacientes (100 lesões) com DRC moderada a grave (TFG < 50 mL/min/1,73 m^2^). O VC médio por procedimento foi de 10,3 ± 7,2 mL. Todas as ICPs foram realizadas com sucesso, sem complicações perioperatórias. Apenas 1 paciente apresentou LRA-AC (1,2%), em comparação com uma incidência prevista de 21,2% ± 10,4% utilizando o escore de Mehran.^26^(p<0,00001). Sekerak et al.^27^em um estudo retrospectivo avaliaram 100 pacientes (TFGe basal mediana: 21,5 mL/min/1,73 m^2^, VC total mediano: 8 mL e VC/TFGe mediano: 0,37) submetidos a ICP VCU guiada por IVUS. De forma semelhante à presente metanálise, os desfechos incluíram LRA-AC, óbito, necessidade de diálise e eventos cardiovasculares adversos maiores (MACE) no acompanhamento de 1 ano. Não houve diferença significativa entre a TFGe pré e pós-procedimento (p = 0,84). Em 1 ano, 8% dos pacientes faleceram, 11% necessitaram de diálise e 33% apresentaram MACE. Allali et al.^28^avaliaram 26 pacientes com DRC avançada submetidos a aterectomia rotacional VCU guiada por IVUS, em comparação com uma população pareada por propensão submetida a ICP padrão. A eficácia foi avaliada pela ausência de LRA-AC após a ICP. A TFGe média foi de 32 ± 17 mL/min/1,73m^2^; o sucesso angiográfico foi alcançado em 100% dos casos; e o VC mediano foi de 12,5 mL. A LRA-AC ocorreu em um paciente e em cinco no grupo de ICP padrão (p = 0,19).

Em um estudo clínico randomizado, Shrivastava et al.^29^avaliaram o desfecho e a segurança a curto prazo da ICP VCU versus ICP convencional em 82 pacientes com síndrome coronariana aguda e alto risco de (LRA-AC) (TFGe < 60 mL/min/1,73 m^2^ e risco pré-procedimento moderado a muito alto de desenvolver LRA-AC, calculado pela calculadora de risco de Maioli).^30^O IVUS foi utilizado em apenas 17% dos pacientes do grupo ICP VCU. Da mesma forma, na presente metanálise, a LRA-AC (desfecho primário) ocorreu mais no grupo de ICP convencional (17,1% vs. 0%; p= 0,012), e não houve diferença significativa nos desfechos secundários de segurança no acompanhamento de 30 dias.

Aggarwal et al.^9^analisaram 463.753 pacientes com DRC basal (TFGe < 60mL/min/1,73m^2^) do registro *National Cardiovascular Data Registry Cath PCI*. Assim como na presente metanálise, o desfecho primário foi a ocorrência LRA-AC. Comparados aos pacientes com baixo VC (> 1 VC /eTFG < 3), aqueles que receberam alto VC (VC/TFGe ≥ 3) apresentaram maior incidência (razão de chances ajustada = 1,36; IC = 1,28 a 1,45) e um estágio mais avançado (razão de chances ajustada = 1,90; IC = 1,80 a 2,00) de LRA-AC, cuja incidência foi semelhante entre os grupos com baixo VC e com VCU (VC ≤ TFGe). A necessidade de diálise foi maior nos pacientes que receberam alto VC (2,8%), em comparação com os grupos com baixo VC (0,8%) e com VCU (0,8%) (p < 0,001). Da mesma forma, Gurm et al.^6^avaliaram o desfecho ICP VCU em 75.393 pacientes. Comparado ao baixo VC (>1 VC/TFGe <3), VCU (VC≤ TFGe, realizado em apenas 13% dos procedimentos) foi associada a incidências significativamente menores de LRA-AC (razão de chances ajustada = 0,682, IC 95% = 0,566–0,821, p< 0,001) e necessidade de diálise (razão de chances ajustada = 0,341, IC 95% = 0,165–0,704, p=0,003). Os benefícios foram mais evidentes em pacientes com alto risco basal previsto de LRA-AC.

Por sua vez, Azzalini et al.^4^avaliaram dados de 111 pacientes (8 no grupo VCU e 103 no grupo de ICP convencional) com DRC avançada (TFGe < 30 mL/min/1,73 m^2^). O VC total foi significativamente menor no grupo ICP VCU (8,8 mL vs. 90 mL; p < 0,001). O protocolo ICP VCU foi bem-sucedido em 88% dos casos, incluindo bifurcação com dois stents, aterectomia rotacional e ICP em oclusão total crônica com suporte mecânico. As incidências de LRA-AC foram de 0% e 15,5% nos grupos ICP VCU e de ICP convencional, respectivamente (p = 0,28).Da mesma forma, Sawant et al.^31^compararam os resultados clínicos da ICP VCU (48 pacientes) versus ICP convencional (152 pacientes). O desfecho primário (LRA-AC ou necessidade de diálise) foi menor no grupo de ICP VCU (2% vs. 15,8%, p=0,01). A incidência de mortalidade por todas as causas foi semelhante em ambos os grupos. Durante o acompanhamento de 6 meses, a redução da função renal foi menor no grupo ICP VCU.

Após o pareamento por escore de propensão, Shibata et al.^21^ avaliaram 50 pacientes divididos em grupos de ICP sem contraste e ICP convencional. Ao contrário da presente metanálise, o desfecho primário foi a incidência de MACE, que foi semelhante entre os grupos (p= 0,95). A ICP sem contraste foi bem-sucedida em todos os procedimentos, sem LRA-AC, necessidade de diálise ou complicações relacionadas ao procedimento.

### Perspectivas futuras

Com o desenvolvimento contínuo de tecnologias e a inovação na área, a ICP VCU será amplamente realizada, ampliando a eficácia das técnicas de revascularização percutânea. Além de pacientes com DRC, técnicas que minimizam o uso de contraste podem ser incorporadas em diversos contextos clínicos, como revascularização percutânea em doenças complexas, dissecções da artéria coronária, choque circulatório, alergias ao contraste, disfunção ventricular esquerda e parada cardíaca extra-hospitalar, síndromes coronárias agudas, intervenções estruturais, incluindo substituição da válvula aórtica por cateter, entre outros. Nesses contextos, a ICP VCU e o uso sistemático de fisiologia e imagem intravascular aumentam a probabilidade de resultados ótimos.^5,7^

### Limitações do estudo

A presente revisão sistemática e metanálise apresenta diversas limitações. A inclusão de poucos estudos, predominantemente não randomizados, introduz um potencial viés, afetando a confiabilidade dos resultados. Estudos com resultados positivos podem ter maior probabilidade de serem publicados. Heterogeneidade relevante (I^2^ > 50%) em todos os desfechos - LRA-AC (I^2^ >96%), necessidade de diálise (I^2^>86%), mortalidade por todas as causas (I^2^>93%), e MACE (I^2^>63%) - reflete variabilidade significativa no desenho do estudo, população e seleção de pacientes, protocolos e técnicas de procedimento, função renal basal, critérios para início da terapia de substituição renal, definições de desfechos, entre outros. Embora tenham sido feitos esforços para mitigar a heterogeneidade, é provável que fatores de confusão residuais persistam. Essas limitações ressaltam a necessidade de ensaios maiores, com poder estatístico adequado, randomizados e controlados, com protocolos padronizados, metodologias e períodos de acompanhamento prolongados, para fortalecer a base de evidências e refinar as recomendações clínicas.

## Conclusões

Em comparação com a ICP convencional, o contraste ultrabaixo em pacientes com DRC mostrou-se seguro, viável e associado a uma redução estatisticamente significativa da lesão renal aguda relacionada ao contraste. Ensaios adicionais, de maior porte, randomizados e controlados, são necessários para confirmar essas descobertas.

## Material suplementar

FIGURAS SUPLEMENTARES
