# Supplementary material for: Intervenção Coronária Percutânea com Volume de Contraste Ultrabaixo versus Intervenção Coronária Percutânea Convencional em Pacientes com Insuficiência Renal Pré-Existente: Uma Revisão Sistemática e Metanálise de Desfechos Clínicos
Source: Arq Bras Cardiol. 2026 May 15;123(4):e20250660. [Article in Portuguese] doi: 10.36660/abc.20250660 (PMC13398812; doi:10.36660/abc.20250660)
Supplement: FIGURAS SUPLEMENTARES [file 0066-782x-abc-123-4-e20250660-suppl01.pdf]

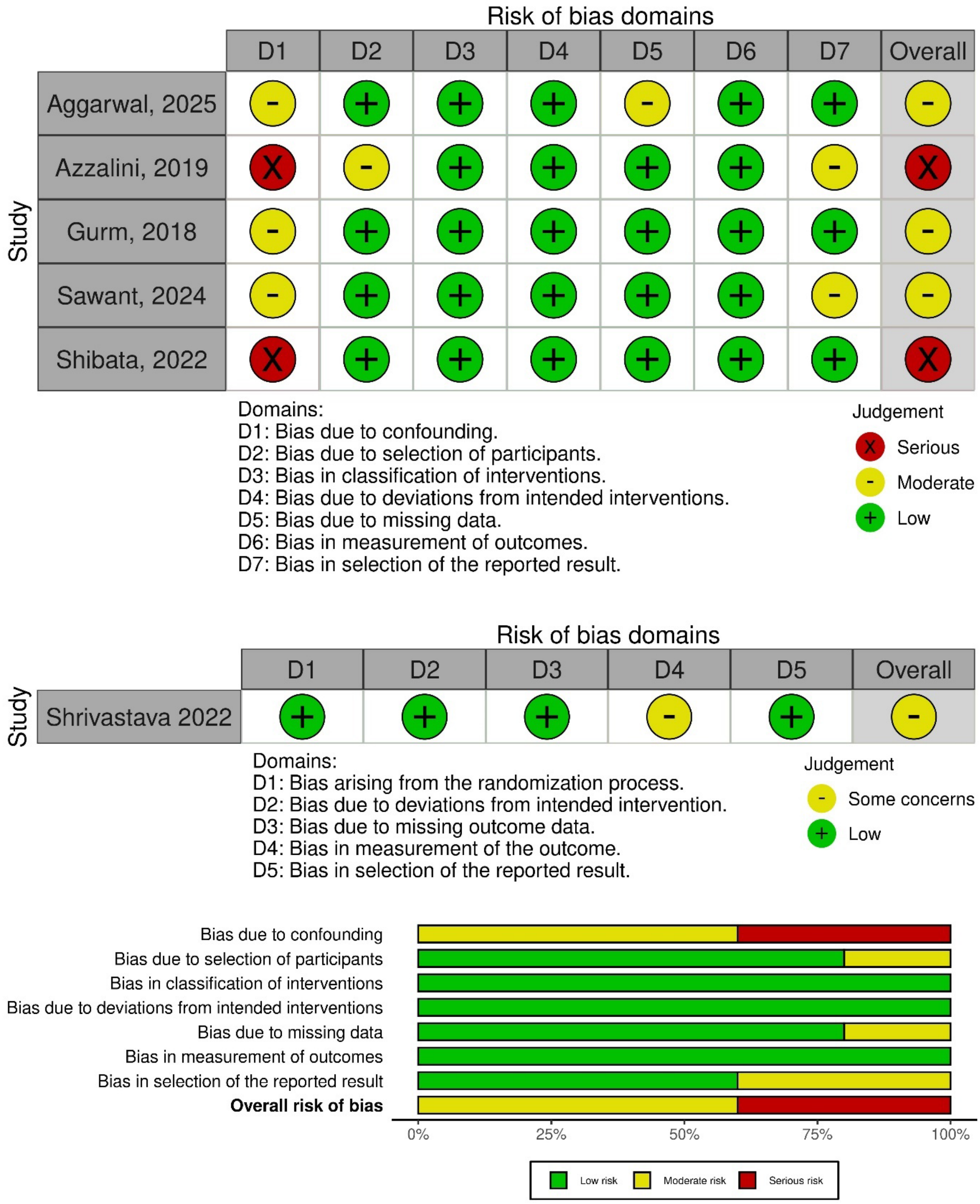

SUPPLEMENTAL FIGURE 1 | Upper and central panels: Risk of bias assessment for included studies, according to ROBINS-I and RoB 2 tools across seven domains. Green (+) indicates low, yellow (−), moderate, and red (X), serious risk of bias. Lower panel: Risk of bias summary across seven domains, showing proportions of low (green), moderate (yellow), and serious (red) risk of bias, according to ROBINS-I and RoB 2 tools.

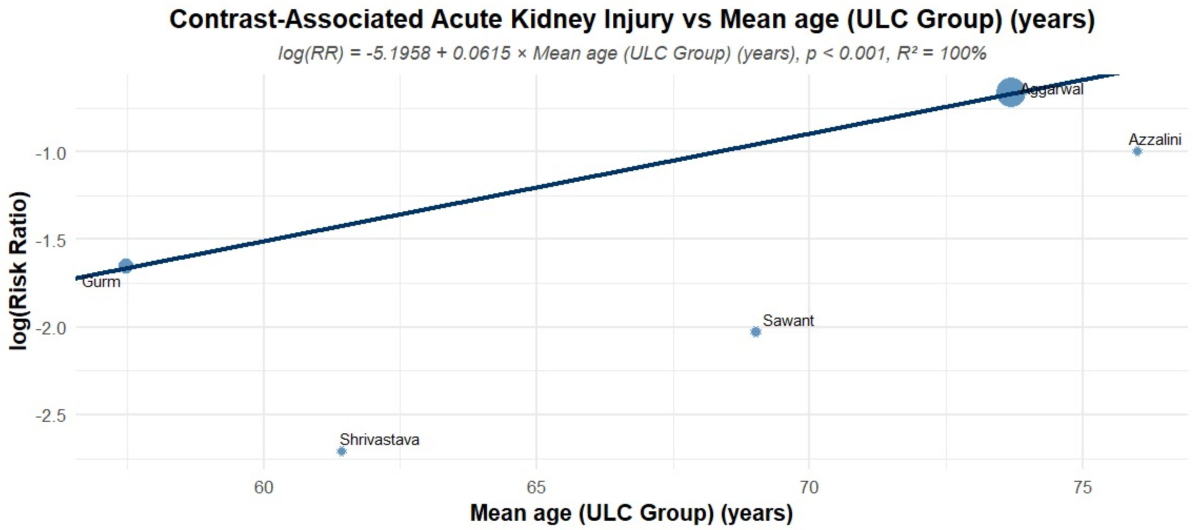

SUPPLEMENTAL FIGURE 2 | Meta-regression analysis: Risk of Contrast-Associated Acute Kidney Injury versus Mean Age for Ultra-Low Contrast Percutaneous Coronary Intervention.

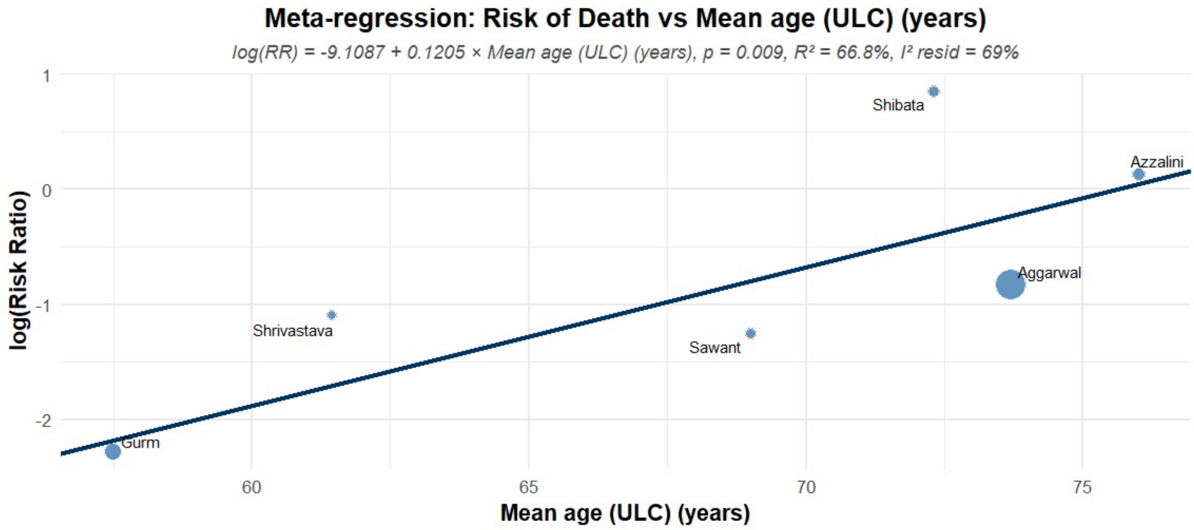

SUPPLEMENTAL FIGURE 3 | Meta-regression analysis: Risk of Death versus Mean Age for Ultra-Low Contrast Percutaneous Coronary Intervention.
