# Supplementary material for: Intervenção Coronária Percutânea com Volume de Contraste Ultrabaixo versus Intervenção Coronária Percutânea Convencional em Pacientes com Insuficiência Renal Pré-Existente: Uma Revisão Sistemática e Metanálise de Desfechos Clínicos
Source: Arq Bras Cardiol. 2026 May 15;123(4):e20250660. [Article in Portuguese] doi: 10.36660/abc.20250660 (PMC13398812; doi:10.36660/abc.20250660)
Supplement: Translation

# Translation: Ultra-Low Contrast versus Conventional Percutaneous Coronary Intervention for Patients with Baseline Renal Impairment: A Systematic Review and Meta-Analysis of Clinical Outcomes

**DOI:** 10.36660/abc.20250660i

**Authors:** Marcos Danillo Oliveira, Ricardo Fonseca Oliveira Suruagy-Motta, Leonardo Dexheimer da Silva, Karlos Daniell Araújo dos Santos, Adriano Caixeta

**Affiliations:** 1 Brazil Departamento de Cardiologia Intervencionista, Universidade Federal de São Paulo, São Paulo, SP – Brazil; 2 Brazil Departamento de Medicina, Universidade Cesmac, Maceió, AL – Brazil; 3 Brazil Departamento de Medicina, Universidade de São Paulo, São Paulo, SP – Brazil; 4 Brazil Departamento de Medicina, Universidade Federal de Roraima, Boa Vista, RR – Brazil

**Keywords:** Contrast Media, Percutaneous Coronary Intervention, Acute Kidney Injury, Systematic Review, Meta-Analysis

## Abstract

**Background:**

Contrast-associated acute kidney injury (CA-AKI) results in increased morbidity and mortality, prolonged hospitalization, and worse clinical outcomes. Prior studies have shown that limiting contrast volume (CV) to less than 3 times the estimated glomerular filtration rate (eGFR) implies a lower risk of CA-AKI, and both efficacy and superior outcomes are associated with ultra-low contrast (ULC) volume coronary procedures (total CV ≤ eGFR).

**Objectives:**

This systematic review and meta-analysis aimed to compare clinical outcomes of ULC versus conventional percutaneous coronary intervention (PCI) in patients with chronic kidney disease.

**Methods:**

The search was conducted in the databases PubMed/MEDLINE, Cochrane, Embase, Scopus, and Web of Science, including all studies reporting clinical outcomes of ULC PCI. The primary efficacy endpoint was the incidence of CA-AKI, and secondary endpoints were need for dialysis, all-cause mortality, and major adverse cardiovascular events (MACE), defined as the composite endpoint of all-cause mortality, non-fatal myocardial infarction, and clinically driven target lesion revascularization.

**Results:**

Six studies met the inclusion criteria and were included in the final analysis, encompassing a total of 274,102 patients. ULC PCI was associated with a statistically significant reduction of CA-AKI (risk ratio=0.27; 95% CI=0.13–0.56; p=0.0004). In turn, ULC PCI did not result in statistically significant reduction of need for dialysis (risk ratio=0.33; 95% CI=0.06–1.72; p=0.186), all-cause mortality (risk ratio=0.48; 95% CI=0.17–1.39; p=0.18), and MACE (risk ratio=0.52; *95% CI= 0.21–1.30; p=0.16).*

**Conclusions:**

ULC, as compared to conventional PCI, in patients with chronic kidney disease, was found to be safe, feasible, and associated with a statistically significant reduction of CA-AKI. Further, larger, well-powered, randomized, and controlled trials are warranted in order to reassure these findings.

## Introduction

Characterized by an impairment in renal function occurring within days after contrast media intravascular administration, in the absence of alternative etiologies,^1,2^ contrast-associated acute kidney injury (CA-AKI) results in increased morbidity and mortality, prolonged hospitalization, and worse clinical outcomes.^3-5^

Over time, improved recognition of risk factors, optimization of contrast agents, and implementation of preventive care resulted in lower rates of CA-AKI.^2^

Appropriate hydration, use of low-osmolar or iso-osmolar contrast media, and contrast volume (CV) minimization are the unique evidence-based resources that have been effective at decreasing CA-AKI.^3,6,7^

Prior studies have shown that limiting CV to less than 3 times the estimated glomerular filtration rate (eGFR) implies lower risk of CA-AKI,^6,8,9^ and both efficacy and superior outcomes are associated with ultra-low contrast (ULC) volume (total CV ≤ eGFR) coronary procedures.^6,10-13^

This systematic review and meta-analysis aimed to compare clinical outcomes of ULC versus conventional percutaneous coronary intervention (PCI) in patients with chronic kidney disease (CKD).

## Methods

This systematic review with meta-analysis followed guidelines and resolutions determined by the Cochrane Handbook^14^ and Preferred Reporting Items for Systematic Reviews and Meta-Analysis (PRISMA) 2020 statement.^15^

In addition, this project has been registered in PROSPERO (https://www.crd.york.ac.uk/PROSPERO/) with protocol number CRD420251108750.

### Search strategy

The search was conducted autonomously by two researchers (RS-M & CF) into the databases PubMed/MEDLINE, Cochrane, Embase, Scopus, and Web of Science, including all studies reporting clinical outcomes of ULC PCI. Boolean operators “AND” and “OR” were applied to combine the terms “Ultra-Low Contrast”, “Percutaneous Coronary Intervention”, and “PCI”. This same strategy was used in all of the databases.

### Eligibility criteria

#### Inclusion criteria

The main question for this systematic review was: “Is ultra-low contrast PCI, as compared to conventional PCI, safe and feasible?” This question was substantiated and formulated using the PICOTT framework,^16,17^ with the following components:

- **Population (P):** adults with baseline CKD (eGFR< 60mL/min/1.73m^2^) undergoing PCI;
- **Intervention (I):** ultra-low contrast PCI (total CV ≤ eGFR for each respective patient);
- **Comparison (C):** conventional PCI (upon standard systematic contrast opacification of coronary arteries for planning, guidance, and assessment of multiple procedural steps and results of PCI, without attempts, techniques, or strategies to minimize total CV);
- **Outcomes (O):**

– Primary endpoint: periprocedural CA-AKI;

– Secondary endpoints: need for dialysis, all-cause mortality, and major adverse cardiovascular events (MACE), defined as the composite endpoint of all-cause mortality, non-fatal myocardial infarction, and clinically driven target lesion revascularization;

- **Type of Study (T):** randomized clinical trials and observational studies;
- **Time (T):** periprocedural up to 6-month follow-up.

#### Exclusion criteria

Exclusion criteria applied for this study were as follows: 1) studies that only addressed ULC strategy without any comparison arm; 2) studies that reported exclusively intravascular ultrasound (IVUS)-guided PCI outcomes; 3) studies without explicit reporting of CV; and 4) studies that did not report quantitative data on CA-AKI or relevant renal outcomes. Additionally, case reports, reviews, conference abstracts, and non-original research were not included in this systematic review and meta-analysis.

## Studies selection

The first screening targeted mainly the title and abstract, without any restrictions on publication date. All articles were imported into the Rayyan platform (https://www.rayyan.]ai/).^18^

Initially, all duplicates were excluded, and then title and abstract screening started. At this stage, all articles that did not directly pertain to the topic of interest were excluded. This step was carried out independently by two reviewers (RS-M and LS); any uncertainties were resolved with the assistance of a third researcher (MDO).

## Endpoints

The primary efficacy endpoint was the incidence of periprocedural CA-AKI.

Secondary endpoints were need for dialysis, all-cause mortality, and MACE, defined as the composite endpoint of all-cause mortality, non-fatal myocardial infarction, and clinically driven target lesion revascularization, periprocedural up to 6-month follow-up.

## Definitions

ULC PCI was defined as PCI performed utilizing a total CV ≤ eGFR for each respective patient.

Zero-contrast (or non-contrast) PCI, a special subset of ULC PCI, was defined as PCI performed utilizing zero mL of contrast dye.

Conventional PCI was defined as PCI performed upon standard systematic contrast opacification of coronary arteries for planning, guidance, and assessment of multiple procedural steps and results of PCI, without attempts, techniques, or strategies to minimize total CV.

According to Kidney Disease Improving Global Outcomes, CA-AKI was defined as an increase in serum creatinine by ≥0.3 mg/dL within 48h after contrast media exposure, or by ≥50% within 7 days.^19^

## Data extraction

The following data were extracted from the selected articles according to the study criteria: authors, year of publication, study location, type of study (randomized clinical trial or observational), sample size, patient demographic characteristics, procedural characteristics, contrast volume, follow-up time, endpoints and outcomes (CA-AKI, need for dialysis, all-cause mortality and MACE), and main results. Two reviewers (RS-M and LS) independently extracted and managed all data, which were recorded in an EXCEL® spreadsheet. Any doubts were resolved with the assistance of a third researcher (MDO).

## Quality assessment

One randomized clinical trial and five observational studies were included in this meta-analysis, and the Risk Of Bias In Non-randomized Studies of Interventions (ROBINS-I) tool^20^was applied, focusing especially on bias related to study design, confounding factors, participant selection, and outcome assessment. Two independent reviewers (LS and RS-M) conducted risk of bias assessments, assigning scores based on established criteria for each tool. Discrepancies between reviewers were resolved through discussion and, when necessary, a third reviewer (MDO) was consulted to achieve consensus. The authors did not use any automated tools during the risk of bias assessment; all grades and evaluations were manually performed by experienced reviewers.

## Statistical analysis

This meta-analysis was conducted following established methodological standards. A random-effects model was employed to account for potential heterogeneity among studies. For binary outcomes, risk ratio (RR) with corresponding 95% confidence interval (CI) were calculated. Statistical significance was defined as p-values < 0.05. Heterogeneity was assessed using the I^2^ statistic, with values > 50% indicating substantial heterogeneity. For such cases, sensitivity analyses were performed using the leave-one-out method, and Baujat plots were generated to assess the influence of each individual study on the overall effect size. Potential publication bias was evaluated through funnel plots constructed for each outcome. All statistical analyses were conducted using R^™^ within the RStudio^™^ environment (version 2024.11.0+488).

## Results

### Study selection

A total of 188 records were identified from PubMed (n = 34), Scopus (n = 39), Web of Science (n = 44), Embase (n = 66), and Cochrane (n = 5). After removing 123 duplicates, 65 records remained for title and abstract screening. Among these, 14 articles were selected for full-text evaluation. Finally, six studies met the inclusion criteria and were then included in the final analysis. This search process is illustrated in Figure 1.

**Figure 1:** – PRISMA flow diagram.

Baseline characteristics of included studies and patients

Table 1 presents the characteristics of the studies included in the current systematic review and meta-analysis, depicting baseline patient information. Most studies demonstrated good balance across the two groups in the number of participants, sex distribution, and age. However, there were some important variations in eGFR values and prevalence of comorbidities.

**Table 1:** – Characteristics of studies included in current systematic review and meta-analysis, depicting baseline patient information

Author, year	Country	Study design	Total number of patients		Male		Mean age		Comorbidities		Chronic kidney disease		Estimated glomerular filtration rate (mL/min/1.73 m^2^)		Total contrast volume (mL)		Number of deployed stents	
-	-	-	Ultra-Low Contrast PCI	Conventional PCI	Ultra-Low Contrast PCI	Conventional PCI	Ultra-Low Contrast PCI	Conventional PCI	Ultra-Low Contrast PCI	Conventional PCI	Ultra-Low Contrast PCI	Conventional PCI	Ultra-Low Contrast PCI	Conventional PCI	Ultra-Low Contrast PCI	Conventional PCI	Ultra-Low Contrast PCI	Conventional PCI
Shrivastava et al.^29^	India	Randomized	41	41	31 (76)	30 (73)	61.44 ± 8.8	60.17 ± 8.17	34 (83) Diabetes / 28 (68) Hypertension	32 (78) Diabetes / 36 (88) Hypertension	26 (63.4)	26 (63.4)	42.20 ± 9.96	41.87 ± 9.34	41.02 (±9.8)	112.54 ± 25.18	1.46 ± 0.66	1.71 ± 0.71
Shibata et al.^21^	Japan	Cohort	50	50	44 (88)	43 (87)	72.3 ± 12.4	70.7 ± 12.0	27 (54.0) Diabetes / 35 (70.0) Hypertension	29 (58.0) Diabetes / 34 (68.0) Hypertension	50 (100)	50 (100)	38.9 ± 15.2	72.6 ± 13.6	4.4 ± 1.3 (IPC)	86.4 ± 38.9 (IPC)	1.14 ± 0.076	1.27 ± 0.07
Sawant et al.^31^	USA	Retrospective	48	152	39 (81)	110 (72)	69 ± 9	68 ± 10	26 (54.2) Diabetes / 44 (91.7) Hypertension	74 (48.7) Diabetes / 147 (96.7) Hypertension	NR	NR	NR	NR	19.17 ± 7.29	147.14 ± 73.55	NR	NR
Gurm et al.^6^	USA	Retrospective	9.857	13.952	6872 (69.7)	8017 (57.5)	57.49 ± 10.85	74.38 ± 10.46	4229 (42.9) Diabetes / 8181 (83.0) Hypertension	5436 (39.0) Diabetes / 12 534 (89.9) Hypertension	NR	NR	NR	NR	106.58 ± 41.19	228.61 ± 83.76	NR	NR
Aggarwal et al.^9^	USA	Cohort	11.621	238.169	7391 (63.6)	139.329 (58.5)	73.7 ± 11.1**	73.7 ± 11.1**	6.461 (55.6) Diabetes / 10.842 (93.3) Hypertension	128.135 (53.8) Diabetes / 219.592 (92.2) Hypertension	11.621 (100)	238.169 (100)	NR	NR	CV/eGFR < 1	CV / eGFR > 3	NR	NR
Azzalini et al.^4^	Italy	Retrospective	8	103	83 (75)		76.0 ± 7.4**		62 (56) Diabetes		8 (100)	103 (100)	19.7 ± 4.4**	24.8 ± 5.9**	9.5 ± 12.7**	96.0 ± 60.7**	NR	NR

Note: Data are presented as mean ± standard deviation, number (no.), or median (interquartile range); PCI: percutaneous coronary intervention; NR: non-reported. *Values reported as N (%) or mean ± standard deviation; **Values transformed to mean ± standard deviation using the method from Wan et al.^*24*^

### Primary outcome

#### Contrast-associated acute kidney injury

The meta-analysis of CA-AKI incidence demonstrated an overall pooled RR of 0.27 (95% CI=0.13–0.56) under a random-effects model, favoring ULC PCI over conventional PCI. The analysis revealed substantial heterogeneity (I^2^=96.7%), indicating significant variability among included studies in terms of design, populations, and procedural protocols. Despite this high heterogeneity, the test for overall effect was statistically significant (Z=-3.52, p=0.0004), supporting a potential protective role of ULC PCI in reducing CA-AKI, as compared to conventional PCI (Figure 2 and Central Illustration).

**Figure 2:** – Forest plot for CA-AKI outcome. The analysis shows overall CA-AKI rates comparing ULC versus conventional PCI across included studies. The black diamond at the bottom represents pooled RR derived from the meta-analysis. CA-AKI: contrast-associated acute kidney injury; ULC: ultra-low contrast; PCI: percutaneous coronary intervention; CI: Confidence Interval; RR: Risk Ratio.

## Secondary outcomes

### Need for dialysis

The meta-analysis of the need for dialysis demonstrated an overall pooled RR of 0.33 (95% CI=0.06–1.72) under a random-effects model, favoring ULC PCI, as compared to conventional PCI, though without statistical significance (Z=−1.32, p=0.186). The analysis revealed considerable heterogeneity (I^2^=86.8%), suggesting substantial differences among included studies in terms of design, baseline renal function, and criteria for renal replacement therapy initiation. Although the point estimate suggests a potential protective effect, the wide CI and high heterogeneity preclude definitive conclusions (Figure 3 and Central Illustration).

**Figure 3:** – Forest plot for the need for dialysis outcome. The analysis shows the overall need for dialysis rates comparing ULC versus conventional PCI across included studies. The black diamond at the bottom represents pooled RR derived from the meta-analysis. ULC: ultra-low contrast; PCI: percutaneous coronary intervention; CI: confidence interval; RR: risk ratio.

### All-cause mortality

The meta-analysis of all-cause mortality demonstrated an overall pooled RR of 0.48 (95% CI=0.17–1.39) under the random-effects model, favoring ULC PCI over conventional PCI, although without reaching statistical significance (Z=-1.35, p=0.18). The high degree of heterogeneity (I^2^=93.7%) indicates substantial variability among included studies, likely due to differences in patient selection, procedural techniques, or endpoint definitions. While the point estimate suggests a potential mortality benefit for ULC strategies, wide CI and marked heterogeneity preclude reliable conclusions (Figure 4 and Central Illustration).

**Figure 4:** – Forest plot for all-cause mortality outcome. The analysis shows the overall mortality rates comparing ULC versus conventional PCI across all included studies. The black diamond at the bottom represents pooled RR.

### Major adverse cardiovascular events

The meta-analysis of MACE showed an overall pooled RR of 0.52 (95% CI= 0.21–1.30) under a random-effects model, suggesting a possible reduction in MACE with ULC PCI, as compared to conventional PCI, although this finding did not reach statistical significance (Z=-1.40, p=0.16). Moderate heterogeneity was observed (I^2^ = 63.8%), reflecting some variability in study protocols, MACE definitions, or patient populations. Despite the direction of effect favoring ULC PCI, CI was wide and crossed unity, precluding firm conclusions (Figure 5 and Central Illustration).

**Figure 5:** – Forest plot for MACE outcome. The analysis shows overall MACE rates comparing ULC versus conventional PCI across included studies. The black diamond at the bottom represents pooled RR derived from the meta-analysis. MACE: major adverse cardiovascular outcome; ULC: ultra-low contrast; PCI: percutaneous coronary intervention; CI: confidence interval; RR: risk ratio.

## Sensitivity analysis

Sensitivity analyses using Leave-one-out, Baujat, and Funnel methods were performed to investigate sources of heterogeneity. Exclusion of Shibata et al.21 reduced heterogeneity to 0% for MACE outcome (Figure 5), and exclusion of Gurm et al.8 reduced heterogeneity to 68% for all-cause mortality (Figure 4) and to 65% for need for dialysis outcomes, respectively (Figure 3).

## Quality assessment

Since one randomized trial was included in this meta-analysis, risk of bias (RoB) assessment was implemented through the Cochrane RoB 2 tool,^22^ following the Cochrane Handbook for Systematic Reviews of Interventions,^14^ which indicated a moderate risk of bias. This was primarily due to unclear randomization methods and some missing outcome data (Supplemental Figure 1).

Additionally, five non-randomized studies were included in this meta-analysis and evaluated by the ROBINS-I tool^20^ (Supplemental Figure 1). Two of these studies exhibited a serious risk of bias, mainly due to baseline differences between the two groups analyzed: there were significant differences in eGFR and substantial disparities in the number of patients in each group. These baseline imbalances can introduce confounding factors that may distort observed effects and compromise internal validity.

Other studies demonstrated a moderate risk of bias in Domain 1 (confounding). While the ROBINS-I tool^20^ provides a structured approach to assess this domain, the inherent risk in non-randomized studies must be considered, and some residual confounding topics can still influence results.

Additional risks identified included unclear study protocols and retrospective allocation of patients, both of which can introduce biases. Unclear protocols can lead to inconsistencies in study implementation, while retrospective allocation can result in selection bias.

## Meta-regression

Regarding the high heterogeneity observed, a meta-regression analysis was performed to explore possible sources of variability. A statistically significant association was found between the mean age of patients in the ULC PCI group and the risks of CA-AKI ( log⁡[RR]=−5.20+0.0615 x mean age  , p < 0.001, R^2^ = 100%, Supplemental Figure 2) and death ( log⁡[RR]=−9.11+0.1205× mean age , p = 0.009, R^2^ = 66.8%, I^2^ resid 69%, Supplemental Figure 3), indicating that age explained the heterogeneity among studies. This finding can be explained by the well-known relationship between age and reduced functional reserve, suggesting that older individuals are more likely to develop CA-AKI and to die, as compared to younger ones.

## Discussion

The main finding of this systematic review and meta-analysis comparing ULC versus conventional PCI in patients with baseline CKD was that ULC PCI resulted in a statistically significant reduction (RR=0.27; 95% CI=0.13–0.56; p=0.0004) of CA-AKI, which is associated with unfavorable prognosis, deleterious clinical outcomes, prolonged hospitalization, and increased mortality.^3-5^

ULC PCI is a contemporary approach that incorporates best practices such as invasive physiology, intravascular imaging, and advanced radiographic tools instead of radiographic contrast.^4^ By combining those tools, with expected safety improvement, both short- and long-term procedural results of these procedures should be better than with conventional angiographic guidance.

Nowadays, several strategies are described to reduce CV during PCI.^5^ Among these approaches, IVUS guidance appears to be the most effective.

The MOZART (Minimizing cOntrast utiliZation With IVUS Guidance in coRonary angioplasTy) study^23^ was the first randomized trial to prove that an intravascular imaging–first strategy can cut contrast use by ~2/3 without harming acute PCI results. Kumar et al.^24^ described their experience with IVUS-guided zero-contrast PCI in 42 consecutive CKD patients (mean eGFR: 30.67 ± 12.26 mL/min/1.73 m^2^, 66 vessels, 21.2% left main PCI). Technical success was 92.4% without any major complications at 3-12-months-follow up. Truong et al.^25^ evaluated a ULC IVUS-guided PCI strategy for 80 patients (100 lesions) with moderate to severe CKD (eGFR <50 mL/min/1.73 m^2^). Mean CV per procedure was 10.3±7.2mL. All PCIs were successfully performed, without periprocedural complications. Only 1 patient experienced CA-AKI (1.2%) as compared with a predicted incidence of 21.2%±10.4% using the Mehran score^26^ (p<0.00001). Sekerak et al.^27^ retrospectively evaluated 100 patients (median baseline eGFR: 21.5 mL/min/1.73m^2^, median total CV: 8mL, and median CV/eGFR: 0.37) who underwent IVUS-guided ULC PCI. Similarly to the present meta-analysis, outcomes included CA-AKI, death, dialysis requirement, and MACE at 1-year follow-up. There was no significant difference between pre- and post-procedure eGFR (p = 0.84). At 1 year, 8% of patients died, 11% required dialysis, and 33% experienced MACE. Allali et al.^28^ assessed 26 patients with advanced CKD undergoing IVUS-guided ULC rotational atherectomy, as compared to a propensity-matched population of standard PCI. Efficacy was assessed by freedom from CA-AKI after PCI. Mean eGFR was 32 ± 17 mL/min/1.73m^2^; angiographic success was achieved in 100%; and median CV was 12.5mL. CA-AKI occurred in one versus five patients in the standard group (p = 0.19).

In a randomized clinical study, Shrivastava et al.^29^ assessed short-term outcomes and safety of ULC versus conventional PCI in 82 acute coronary syndrome patients at high risk for CA-AKI (eGFR < 60mL/min/1.73m^2^ and moderate to very high pre-procedural risk of developing CA-AKI, as calculated by the Maioli risk calculator).^30^ IVUS was used only in 17% of patients of the ULC PCI group. Likewise, the present meta-analysis, CA-AKI (primary outcome) occurred more in the conventional PCI group (17.1% vs. 0%; p = 0.012), and there was no significant difference in secondary safety outcomes at 30-day follow-up.

Aggarwal et al.^9^ analyzed 463,753 patients with baseline CKD (eGFR < 60mL/min/1.73m^2^) from the National Cardiovascular Data Registry CathPCI registry. As in the present meta-analysis, the primary outcome was the occurrence of CA-AKI. Compared to low CV (>1 CV/eGFR <3), patients who received high CV (CV/eGFR ≥3) had a higher incidence (adjusted odds ratio=1.36; CI=1.28 to 1.45) and a higher stage (adjusted odds ratio=1.90; CI=1.80 to 2.00) of CA-AKI, whose incidence was similar between low CV and ULC (CV ≤ eGFR). The need for dialysis was higher in patients who received high CV (2.8%), as compared to low CV (0.8%), and ULC (0.8%) (p < 0.001). Similarly, Gurm et al.^6^ assessed outcomes of ULC PCI among 75,393 patients. Compared to low CV (>1 CV/eGFR <3), ULC (CV ≤ eGFR, undertaken in only 13% of procedures) was associated with significantly lower incidences of CA-AKI (adjusted odds ratio = 0.682, 95% CI=0.566–0.821, p < 0.001) and need for dialysis (adjusted odds ratio = 0.341, 95% CI= 0.165–0.704, p=0.003). Benefits were most evident in patients with high baseline predicted risk of CA-AKI.

In turn, Azzalini et al.^4^ evaluated data from 111 patients (8 in the ULC and 103 in the conventional PCI groups) with advanced CKD (eGFR < 30 mL/min/1.73 m^2^). Total CV was markedly lower in the ULC PCI group (8.8mL vs 90mL; p < 0.001). ULC PCI protocol was successful in 88% of the cases, including two-stent bifurcation, rotational atherectomy, and mechanically supported chronic total occlusion PCI. The incidences of CA-AKI were 0% and 15.5% in the ULC and conventional PCI groups, respectively (p = 0.28). Likewise, Sawant et al.^31^ compared clinical outcomes of ULC (48 patients) versus conventional PCI (152 patients). The primary endpoint (CA-AKI or need for dialysis) was lower in the ULC PCI group (2% vs. 15.8%, p=0.01). The incidence of all-cause mortality was similar in both groups. During the 6-month follow-up, the decrement in renal function was lower in the ULC PCI group.

After propensity score matching, Shibata et al.^21^ assessed 50 patients divided into zero-contrast PCI and conventional PCI groups. Unlike the present meta-analysis, the primary endpoint was the incidence of MACE, which was similar between the groups (p = 0.95). Zero-contrast PCI was successful in all procedures, with no CA-AKI, need for dialysis, or procedural complications.

### Future perspectives

With the ongoing development of technologies and further innovation in the field, ULC PCI will become widely performed, which will broaden the effectiveness of percutaneous revascularization techniques. Apart from patients with CKD, contrast-sparing techniques might be incorporated in several clinical settings, like complex disease percutaneous revascularization, coronary artery dissections, circulatory shock, contrast allergies, impaired left ventricular function, out-of-hospital cardiac arrest, acute coronary syndromes, structural interventions, including transcatheter aortic valve replacement, among others. In such contexts, ULC PCI and systematic use of physiology and intravascular imaging enhance the likelihood of optimal results.^5,7^

### Study limitations

The present systematic review and meta-analysis have several limitations. The inclusion of a few and predominantly non-randomized studies introduces potential bias, affecting the reliability of results. Studies with positive findings may be more likely to be published. Relevant heterogeneity (I^2^>50%) in all outcomes - CA-AKI (I^2^>96%), need for dialysis (I^2^>86%), all-cause mortality (I^2^>93%), and MACE (I^2^>63%) - reflects significant variability in study design, patient population and selection, procedural protocols and techniques, baseline renal function, criteria for renal replacement therapy initiation, endpoints definitions, among others. While efforts were made to mitigate heterogeneity, residual confounding factors likely persist. These limitations underscore the need for further larger, well-powered, randomized, and controlled trials, with standardized protocols, methodologies, and extended follow-up periods, to strengthen evidence base and refine clinical recommendations.

## Conclusions

Ultra-low contrast, as compared to conventional percutaneous coronary intervention, in patients with CKD, was found to be safe, feasible, and associated with a statistically significant reduction of contrast-associated acute kidney injury. Further, larger, well-powered, randomized, and controlled trials are warranted in order to reassure these findings.

## Supplemental Materials

SUPPLEMENTARY FIGURES
